# Understanding Bacteriophage Specificity in Natural Microbial Communities

**DOI:** 10.3390/v5030806

**Published:** 2013-03-11

**Authors:** Britt Koskella, Sean Meaden

**Affiliations:** BioSciences, University of Exeter, Cornwall Campus, Tremough, TR10 9EZ, UK; E-Mail: sm341@exeter.ac.uk

**Keywords:** coevolution, infection genetics, phage therapy, kill the winner

## Abstract

Studying the coevolutionary dynamics between bacteria and the bacteriophage viruses that infect them is critical to understanding both microbial diversity and ecosystem functioning. Phages can play a key role in shaping bacterial population dynamics and can significantly alter both intra- and inter-specific competition among bacterial hosts. Predicting how phages might influence community stability and apparent competition, however, requires an understanding of how bacteria-phage interaction networks evolve as a function of host diversity and community dynamics. Here, we first review the progress that has been made in understanding phage specificity, including the use of experimental evolution, we then introduce a new dataset on natural bacteriophages collected from the phyllosphere of horse chestnut trees, and finally we highlight that bacterial sensitivity to phage is rarely a binary trait and that this variation should be taken into account and reported. We emphasize that there is currently insufficient evidence to make broad generalizations about phage host range in natural populations, the limits of phage adaptation to novel hosts, or the implications of phage specificity in shaping microbial communities. However, the combination of experimental and genomic approaches with the study of natural communities will allow new insight to the evolution and impact of phage specificity within complex bacterial communities.

## 1. Introduction

Whether found in the soil [1,2], the leaf [3,4], the ocean [5,6], or the human body [7,8], microbial communities are proving to be more dynamic and diverse than could have been predicted. This incredible diversity is seen both within environments and among environments [7], but how it is generated and maintained is unclear. Early ideas about microbial diversity posited that “Everything is everywhere, but the environment selects [9].” However, this view of the microbial world remains hotly debated [10]. Much of the work testing this tenet has focused on the abiotic environment, such as salinity [11] or soil types [12], but the biotic environment is likely to be just as, if not more, important in shaping selection on microbial populations. Bacteria in any given environment face strong selection pressures from other microbes, predators, viruses, and in the case of bacteria living within another organism, the host immune response. In contrast to the abiotic environment, biotic “environments” have the potential to evolve in response to any changes in the microbial community, making them highly dynamic and capable of driving divergence among populations [13,14]. Bacteriophages (phages) represent perhaps the most ubiquitous of these biotic drivers [15,16,17]. To understand the role of phage-mediated selection in generating diversity, however, we need good insight into how specific phages are to their bacterial hosts. 

For phages to alter the composition of a microbial community, there must exist a degree of specificity such that some hosts are more resistant to local phages than others or are better able to respond to phage-mediated selection. There is clear evidence that not all bacteria are infected by all phages, and indeed that most phages can only infect a subset of bacterial species (Table 1; [18]), but our understanding of phage host range is far from complete. Can phages easily adapt to infect new bacterial types as they become common? Can the same phage lineage shift from one bacterial species to another? These questions are far from new [19], but the development of recent techniques and the power of comparative genomics are moving us towards more satisfying answers. Experimental evolution (Box 1) provides one powerful approach to address these knowledge gaps, as the bacteria-phage interaction can be observed in the absence of other abiotic or biotic selection pressures; as such it has offered key advances in our understanding of the evolution of bacterial resistance to phages and reciprocal adaptations of phages to overcome such resistance. However, there are many reasons that the outcome of coevolution in a test tube might not be predictive of coevolution in nature, given the added biotic and abiotic complexity of most microbial ecosystems. For example, although experimental evolution studies have almost exclusively focused on phage adaptation within a population of one, or at most a few, bacterial species, most bacterial communities are highly rich, and therefore most bacterial species to which phage are adapting are rare. A comparison of culture-independent sequencing studies of microbial communities from the leaf surface, soil, atmosphere and the human body shows that the most dominant species in each given community represents a mere 2–5% of sequences [20]. Given the heterogeneity and diversity of these microbial communities, it is unclear how a phage with narrow host range could evolve and be maintained.

For virulent bacteriophages, *i.e.*, those that reproduce within and then lyse their host cells, success depends on the chance event of encountering a susceptible host cell in the environment, and is most certainly reduced as a function of community diversity, dispersal, and exposure to the harsh conditions outside of a cell. It makes intuitive sense, therefore, that those phages with a larger host range should be at an inherent advantage. The data gathered so far, however, do not clearly support this intuition. First, many phages seem to be specific to a single bacterial species, and are often specific to only a few strains within that species [21,22,23] (Table 1). Second, there is building evidence that phages are “locally adapted” to their bacterial hosts [3,24], indicating a degree of specialization to common bacterial strains or species in a given population. Third, although phages do tend to increase their host range during the initial stages of coevolution, there is evidence that this expansion is short-lived [25]. The underlying mechanisms of phage infectivity and bacterial resistance are of course key to the evolution of phage host range [22,26], and have been the focus of extensive review elsewhere [21,27]. The data make it clear that phage infectivity is a complex function of adsorption [28], structural change of both host and phage [29], transport of nucleic acid into the bacterial cell, and avoidance of degradation once inside the cell [30], and is thus a result of both phage and bacterial phenotype. In addition, host susceptibility/resistance to phage can be determined by plasmids hosted by the bacterial cell [31,32]. This form of phage-plasmid interaction could lead to broad phage host range due to horizontal transfer of the plasmid among bacterial species within a community. A more thorough understanding of these interacting mechanisms will allow us to better predict the potential for host range expansion/contraction and therefore the effect of phages on microbial communities under both natural and therapeutic settings.

The more general question of why parasites specialize is of course not specific to bacteria-phage interactions, and we can apply much of the current coevolutionary theory to understanding the evolution of phage specificity. Many phages act as obligate parasites, as they are both unable to reproduce outside of their host cells and require cell lysis to transmit, thus killing their hosts. However, we acknowledge that, although virulent phages are obligate killers, other phages integrate into the host genome and their fitness relies on host reproduction. In these cases, the acquisition of a prophage can confer beneficial phenotypic change to the bacterial hosts, and therefore this latter relationship acts more synergistically than antagonistically. In either case, the question of host range for phages that are in the lytic cycle, and being transmitted among cells can be broken into two parts: first, the specificity of host resistance against infecting parasites; and second, the specificity of parasites on different hosts. It is often difficult to tease these two processes apart, but a recent review suggests that the failure to infect “nonhost” species (*i.e.*, those not considered to be hosts for the pathogen in question) may be the result of pathogen evolution leading to specialization on its own source host species and not the result of host evolution for resistance [33]. By reviewing studies across many host-parasite systems, the authors find a general trend towards decreasing parasite infection success on hosts of increasing genetic distance from the focal host. It remains to be determined whether this pattern is ubiquitous for bacteria-phage interactions, especially given the broad host ranges of some phages (Table 1).

The most supported evolutionary argument for why parasites specialize on given hosts, despite the clear advantage of a broad host range, is that there exists a trade-off between fitness and the breadth of parasite infectivity or host resistance [34]: In other words, the idea that “a jack of all trades is a master of none.” This can be explained either by antagonistic pleiotropy, a situation where an adaptation that is advantageous in one host is deleterious in another, or else by selection for a less efficient but more general mechanism of infection. Support for this trade-off has been found for phage ϕ2, where individuals with broader host range within populations of its bacterial host, *Pseudomonas fluorescens,* were shown to pay a cost for this increased breadth relative to phages with narrow host ranges [35]. Specific evidence of antagonistic pleiotropy has also been found; during experimental host range expansion of phage ϕ6, spontaneous mutants able to infect novel hosts were found to be less infective to their native hosts in seven out of nine cases [36]. These trade-offs are also likely to be common in host populations. Indeed, recent results from experimental evolution (see Box 1) of *Prochlorococcus* hosts and their associated phages demonstrate that resistance to one phage genotype often came with the added cost of increased susceptibility to another phage genotype [37]. Similarly, experimental evolution of *P. syringae* in either single phage or multiple phage environments shows that bacteria evolved with multiple phages paid a higher cost of resistance than those evolving with single phages [38].

**Box 1. Experimental evolution of phage specificity.**

**Figure 1:**
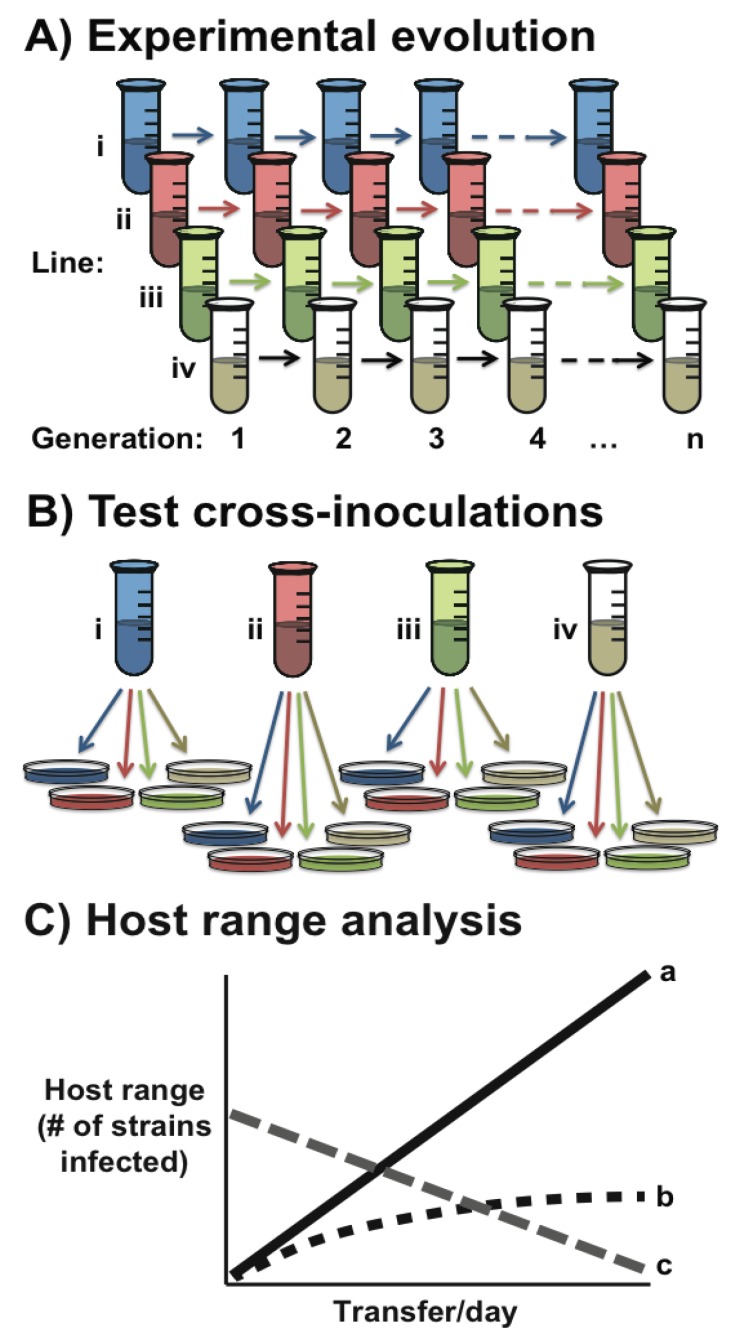
Illustrative example of experimental evolution of phage host range, where: (**A**) independent lines of genetically identical phage populations are propagated under different treatment regimens (e.g., different bacterial host species); and then (**B**) tested for infectivity on focal and alternate hosts. (**C**) Outcomes of these experiments might be a directional change towards increased host range over time (a), an initially increasing but then stable host range, perhaps indicative of coevolutionary response by the host population (b), or a decrease in host range associated with antagonistic pleiotropy during specialization on the focal host (c).

Experimental evolution of bacteriophage specificity has offered some key insights into the underlying process, evolutionary consequences, and fitness costs of host range expansion. The power of this method is that it allows replicate lines, started with genetically identical phages, to be passaged on homogeneous or heterogeneous hosts populations under a range of conditions (such as density and resources) for many thousands of generations (Figure 1). During this time, both the host bacterium and the phage can be frozen in time and resurrected at the end of the experiment, at which point [39] the fitness of evolved phages can be compared directly to both the ancestral types and phages experiencing a different selection regimen.

This approach has been used to demonstrate a number of key features of phage specificity, and has gone some way in explaining both when and how phage host range is likely to expand. First, in terms of range expansion within a host population (*i.e.*, the evolution of “generalist” phages capable of infecting more genotypes of a given bacterial species), there is evidence that phage ϕ2 is more likely to increase its host range during experimental coevolution with its bacterial host, *P. fluorescens*, than when the bacterial population is held constant [35]. Similar results were found in coevolving populations of phage SBW25ϕ2 and *P. fluorescens* [40]. Furthermore, the emergence of evolved “generalist” phenotypes of both bacteria and phages during experimental coevolution has been demonstrated both in a marine cyanobacteria and cyanophage system [5] and in a *P. fluorescens* and phage SBW25ϕ2 system [41]. Second, in terms of host range expansion to novel hosts, experimental evolution has provided evidence that phage ϕ6 populations are more likely to evolve expanded host range when there is strong competition for hosts (*i.e.*, when the focal host is rare in a population) [42]. It has also been shown that during the early stages of such a host shift, the likelihood of successful adaptation to a novel host is increased when contact with the native host is maintained, as this prevents extinction of the phage [43].

An experimental coevolution approach can also be taken to identify the mutations underlying gains or losses of host types. For example, host range expansion of phage ϕ6 to a novel host was found to be associated with a single nucleotide change [44]. Furthermore, phages experimentally coevolved with *P. fluorescens* hosts evolved increased host range over time and the phage genotypes with the broadest host ranges were found to have the most nonsynonymous amino acid changes, especially in the phage tail fiber gene [45]. Finally, experimental evolution of phage λ on populations of *Escherichia coli* that had lost the receptor used for phage attachment were found to evolve the ability to infect the bacterial host via a novel receptor following the spread of key precursor mutations, suggesting that phage host shifting can occur via entirely new innovations [46]. Clearly, the power of experimental evolution in understanding phage host range has not been fully exploited and moving forward this approach will offer further insight to the evolution of phage specificity in complex bacterial communities, fitness trade-offs between broad and narrow host ranges, and the potential limits of host shifting among phages.

### 1.1. The Structure of Bacteria-Phage Interaction Networks

Studies of phages in natural populations have confirmed that, like many other parasites, phages are well adapted to their local host populations [3,24,47]. However, whether individual phages are specialized on certain genotypes/species, the frequencies of which differ across space, or whether coevolution is driving among-population divergence remains to be determined. Importantly, phage specificity can only be meaningfully evaluated within a culturable reference set of host and/or parasite genotypes; and a different reference panel of host or pathogen genotypes may reveal different levels of specificity. Choosing an appropriate reference set is often challenging, as the interaction networks and species ranges of most phages is not known, but it is of central importance to choose a panel that is biologically meaningful and/or informative to the predictions being tested.

Those studies that have looked at host range of individual phages from the environment demonstrate large variation in specificity, both within and across bacterial species. In fact, some phages that appear to be “generalist,” in the sense that they can infect bacteria spanning genera, fail to infect a subset of strains or species within genera (Table 1). This apparent inconsistency, which is likely the result of both specific phage adaptations and the subsequent evolution of bacterial resistance in some lineages, including via transfer of plasmids, makes it difficult to decipher specific rules regarding phage host range. For example, populations of *Candidatus* isolated from two separate sludge bioreactors were found to differ primarily in genomic regions encoding phage defense mechanisms, despite global dispersal of the strains among the two sites [48]. It is only when many of these studies are compared and datasets are compiled that clear patterns emerge. A recent meta-analysis of the statistical structure of bacteria-phage interactions shows that the infection networks are non-random and are typically nested [18]. This means that the highly resistant bacterial strains/species are only infected by broad host range phages, whereas the highly susceptible bacteria are infected by phages with narrow to broad host ranges. The observed lack of modularity may be suggestive of a true continuum of phage host range. Alternatively it might reflect the fact that most studies included in the analysis examine interactions either within a single bacterial species (*i.e.*, across multiple strains/genotypes) or between phages from one environment on bacteria from entirely different communities, habitats, and even continents. To determine the statistical structure of bacteria and phage communities in nature requires analysis of phage host range on representative bacterial hosts from the same local habitat.

### 1.2. The Evolutionary Implications of Phage Host Range

Given that phages are relatively specific, are capable of rapid adaptation, and are often obligate killers of their host cells, they can impose strong selection on bacterial populations and shape microbial communities. The “Kill the Winner” hypothesis posits that phages adapt to and preferentially infect the lineages of bacteria with the highest frequencies in the population, for example, those with higher metabolic fitness. Evidence for this hypothesis has been collected in a number of ways, including experimental coevolution of phages and bacteria in a test tube [49], monitoring of population change over time [50,51], and using metagenomic approaches [16]. Killing the winner is a form of negative frequency-dependent selection, as bacterial fitness is an indirect function of its frequency in the community. Specifically, bacterial species that are rare and free from phage attack will increase in frequency until the point at which an infective phage is introduced, either via mutation or migration, and spreads through the population. At this point, the common bacterial species will be at a relative disadvantage and may decrease in frequency.

Clearly, this type of dynamic is only possible if specificity underlying infection exists, but it also requires a time lag during which previously rare bacterial species can increase in frequency and remain free from phage attack. The length of this lag, *i.e.*, the time it takes for a phage mutant to arise by mutation or immigration, will dictate how common bacterial species can become before being targeted by coevolving phages. There is also likely to be a lag in the time it takes for a rare phage genotype to increase in frequency as its respective host becomes common, especially in populations where bacterial densities, and therefore rate of encounter, are low. Extending this theory to bacteria-phage interactions, we might predict that oscillatory dynamics should be more pervasive in relatively closed microbial communities, such as the human gut, than in highly connected communities, such as the ocean. This can be extended to predict that microbial communities with higher gene flow of bacteria and/or phages should show greater species evenness than closed communities, where frequencies are fluctuating over time. The data on phage infection of common bacterial species remains scarce, but the evidence we do have is in line with the above prediction. Estimates from marine communities suggest that cyanobacterial cell lysis by cyanophages ranges from a mere 0.005% to 3.2% per day, depending on the season [52]. Similarly, for bacterial isolates collected from the surface of tree leaves, a habitat that is open to constant immigration, only 3% of bacteria were found to be susceptible to local phages. This is in stark contrast to the interior of those same leaves, a more protected and closed microenvironment, where 45% of bacterial isolates were found to be susceptible to local phages [3]. It is important to note, however, that a number of differences exist among these habitats beyond the potential for immigration [53]. A comparable result was found in natural soil samples, where 33 to 40% of bacteria could be lysed by phages from the same sample [24]. Given the paucity of studies that have explored bacteria-phage dynamics in nature, the ubiquity of phage-mediated negative frequency-dependent selection remains unclear. Future studies exploring the natural prevalence of phage infection, coevolutionary dynamics of bacteria and phages over time, and the evolution of phage host range in either natural or experimental communities are still needed to predict how phages influence bacterial communities.

### 1.3. The Applied Implications of Phage Host Range

In addition to their potential role in shaping bacterial community composition, phages are key players in shaping the evolution of bacterial genomes [54]. As lytic bacteriophages reproduce within the host and reassemble, bacterial chromosomal DNA can be inadvertently packaged into the viral capsid along with the viral DNA. This mistake will lead to *generalized* transduction (in contrast to *specialized* transduction by prophages) and can move chromosomal DNA from one bacterial host to another. During this movement among hosts, phages can transfer genes encoding toxins or virulence factors, and thus critically alter the bacterial phenotype. For example, phage-mediated transfer of pathogenicity islands between *Listeria monocytogenes* and *Staphylococcus aureus* has been demonstrated in raw milk [55] and phage-mediated transfer of antibiotic resistance has been demonstrated among species of *Enterococcus* [56]. It is increasingly clear that transduction can occur across distantly related bacterial species, and even the seemingly highly conserved 16S rRNA gene has been found within the genome of a broad host range transducing phage [57]. Thus understanding phage specificity among bacterial strains and species is key to predicting potential movement of genes across bacterial species and habitats, and thus the potential emergence of novel pathogens.

An understanding of phage specificity is also central to predicting the success and consequences of phage therapy, *i.e.*, the use of phage or cocktails of phages to control the growth and/or virulence of pathogenic bacteria. The utility of phage therapy is often called into question because of the apparent specificity of phages [58]. However, this specificity is also a clear advantage of phage therapy over more general treatments, such as antibiotics, since the non-target bacterial populations should remain relatively undisturbed. The first steps in testing the benefits of a potentially therapeutic phage are to test a) whether the phage is too specific to be effective against the standing strain variation of a pathogen in a host population and b) the likelihood that the phage will affect other non-pathogenic bacteria, either immediately due to a large host range or over short evolutionary timescales as the phage evolves. For example, recent work from silage of dairy farms found a great deal of strain-to-strain variation in susceptibility of the food-borne pathogen, *L. monocytogenes,* to phages collected from silage. They tested the host range of 114 listeriaphages and found that 12% of these phages had narrow host ranges and could infect fewer than half of the strains tested, representing the nine major serotypes of *L. monocytogenes.* However, another 29% of the phages were capable of infecting nearly all of the strains tested, suggesting that these phages would be good candidates for therapeutic control of the pathogen [59]. Furthermore, given the ease of full genome sequencing, it is now possible to scan the phage genome for virulence factors and known toxin-encoding genes to ensure the phage will not act to increase the harm caused by a given pathogen.

## 2. Results and Discussion

### 2.1. Specificity within a Natural Phyllosphere Environment

The microbial community within eukaryotic hosts is a relatively closed system that holds the potential for long periods of uninterrupted bacteria-phage coevolution, especially if the hosts are long-lived. Recent work examining bacteria and phages from the horse chestnut phyllosphere (*i.e.*, the above-ground, aerial habitat of the plant) has demonstrated strong local adaptation of phages to bacteria collected from the same leaf [3]. Given that the culturable bacterial communities found within these leaves differed among the trees sampled, it is unclear whether this result demonstrates adaptation of multiple phages to common bacterial strains (which differ among populations) or whether it suggests phage adaptation to infect the common bacterial species within a given community. In other words the result could indicate species sorting according to infection success or it could suggest coevolution of bacteria and phages within each population. One way to tease these two possibilities apart would be to examine specific phage clones from each population and measure their host ranges both within the bacterial community from which they were isolated and from other communities.

**Table 1:** Examples of phage specificity from natural populations. Habitat refers to the environment from which the samples were selected, and host to the bacterial species used to first visualize the phage. For each study, the number of phages tested is reported and the host range of these phages is described depending on whether the phages were able to infect bacteria from multiple species and/or multiple genera. Finally, we report whether there was variability in phage infectivity on different strains/genotypes within a single bacterial species. In all cases, “n/a” is reported when the phages were not tested in a way that allowed for a given comparison. Two cases show both within-species specificity and an ability to infect multiple species, and these are highlighted in bold to emphasize the difficulty in describing a given phage as “generalist” versus “specialist.” Note that this table is for illustrative purposes and is not exhaustive. For a formal meta-analysis of bacteria-phage infection networks, see recent review by Flores and coauthors [60].

			Host range			
Habitat	Host	# Phages tested	Multi-species	Multi-genus	Within-species specificity	Reference
Rhizosphere	Pseudomonas	5	4	0	n/a	Campbell *et al*. 1995 [61]
Sewage	Multiple hosts	11	n/a	11	n/a	Jensen *et al.* 1998 [62]
Industrial	Leuconostoc	6	0	0	Yes	Barrangou *et al*. 2002 [63]
Marine	Vibrio	13	**10**	n/a	**Yes**	Comeau *et al*. 2005 [64]
Soil	Burkholderia	6	**6**	n/a	**Yes**	Seed and Dennis 2005 [65]
Effluent	Salmonella	66	n/a	0	Yes	McLaughlin *et al*. 2006 [66]
Marine	Cellulophaga	46	0	0	Yes	Holmfeldt *et al*. 2007 [67]

**Figure 2:**
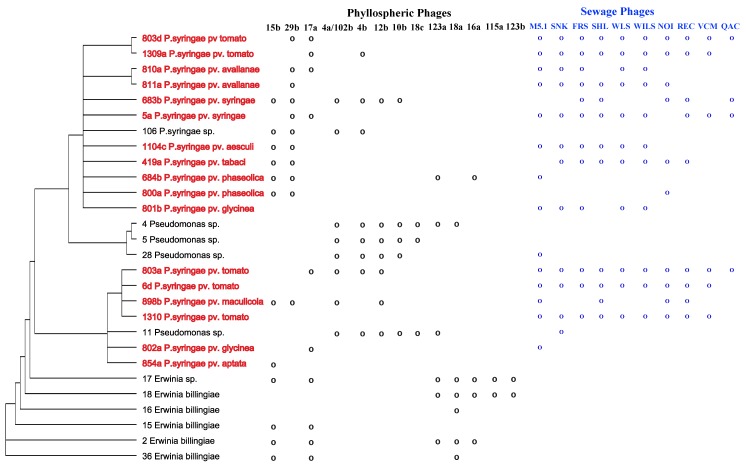
Neighbor-joining tree showing the phylogenetic relationships among bacteria used in this study and their susceptibility to bacteriophages from the phyllosphere (black) or from sewage (blue). Trees are based on 16S rRNA gene sequences (∼800 bp). Bacterial isolates in red have been classified previously to the pathovar level. Phages 4a and 102b had identical host ranges, despite being isolated from separate leaves, and their profiles have thus been collapsed into one.

As a first step towards this, we randomly selected 144 culturable bacterial isolates from across the eight trees sampled in the original experiment [3] and inoculated each isolate with a dilution series of the sympatric phage community (*i.e.*, filtered leaf homogenate, note that no enrichment procedure was used). For the 14 bacteria that were susceptible to their local phages, a single phage plaque was isolated and re-inoculated into an overnight culture of the bacteria it was able to infect. These co-cultures were then filtered, creating a high titer phage inoculum made up of a single phage clone. All 14 of these phages were cross-inoculated onto each of the 144 original bacterial host isolates, as well as 17 previously characterized *P. syringae* isolates representing nine different pathovars, to determine host range (Figure 2). In addition, we measured the susceptibility of each bacterium in the reference panel to ten phages that were previously collected from sewage and enriched on *P. syringae* pathovar tomato. It is important to note that the host range examined here represents the phage’s *plaquing* host range (*i.e.,* the range of hosts a given phage can successfully infect and lyse in soft agar [21]) and may be an underrepresentation of its *productive* (*i.e.*, phage-producing) host range. We sequenced ≈800 bp of the 16S ribosomal RNA region from all hosts that were found to be susceptible to phage. Prior to sequencing, bacterial isolates were grown in KB broth overnight. These overnight cultures were then diluted 1:5 in PCR grade water and used as PCR template (5 μL) in reactions with universal 16S primers 515f (5-GTGCCAGCMGCCGCGGTAA-3) and 1492r (5′-GGTTACCTTGTTACGACTT-3′) [68]. Diluted PCR products (50 ng/μL) were sequenced in both directions, and then aligned and compared against the NCBI database using Geneious (v2.5) software. Individual isolates were assigned to a given genera and species when possible according to the top BLAST hits associated with the sequence (with an e-score of 0.0). Geneious was used first to align the sequences (using MUSCLE, multiple sequence comparison by log-expectation [69]) and then a consensus neighbor-joining tree was assembled with pairwise distances calculated using the “Jukes-Cantor” formula.

We examined the resulting network of bacteria-phage interactions using a recently described method [70]. Relative to the null model, the network shows very little evidence of nestedness, in that the phages with broad host range do not tend to infect the more resistant hosts (the network is only 1% more nested than expected by chance). Instead, there is evidence that the network is highly modular; the network shows 82% of interactions occur between isolates from the same module relative to a null model of 66%. This is suggestive that the pattern of local adaptation observed previously [3] is indicative of many phages each coevolving with a subset of the bacterial host community. However, given that the phages and bacteria were collected from across eight separate microbial “populations,” represented by eight different tree hosts [3], a larger analysis will be required to confirm that the modularity represents bacteria-phage coevolution at small spatial scales or whether it indeed indicates that these natural microbial communities are harboring phages with largely non-overlapping host ranges. The phages examined clearly fall across the continuum of “generalist” to “specialist” (note that none were restricted to a single bacterial isolate), but most are restricted to infecting fewer than one third of the bacterial isolates. Of the 13 phages isolated from the phyllosphere, 5 are capable of infecting both *Pseudomonas* and *Erwinia* species and most are capable of infecting multiple pathovars within *P. syringae* (Figure 2). Interestingly, the phages isolated from sewage had a relatively wider host range across the *Pseudomonas* isolates, infecting a mean of 10.9 (SD = 3.21) hosts, than did the phages from the phyllosphere, infecting a mean of 7.57 (SD = 3.18) hosts. This is not surprising, as the method of searching for phage in one environment using a host bacterium from another is likely to bias the resulting isolates towards more “generalist” phages. Finally, we found two phages collected from the horse chestnut leaf that are capable of infecting a previously characterized strain of *P. syringae* pathovar aesculi (Pae), the causal agent of bleeding canker disease in horse chestnut trees. These phages were not, however, capable of infecting the other three strains of Pae we tested, suggesting that there is variation in susceptibility of Pae strains to phages despite the relatively low genetic diversity typically found among isolates of this rapidly emerging pathogen [71].

### 2.2. The Importance of Dose in Measuring Specificity

In addition to taking into consideration the appropriate reference panel when measuring phage specificity, it is also necessary to take into account that bacterial sensitivity is likely to depend critically on both phage titer and test conditions. Most studies examining the specificity of parasites within their local communities [18,72,73,74] treat infectivity as a binary trait and examine bacterial sensitivity at a single phage titer and without taking into account environmental effects on the outcome of the interaction. This is often a necessary step given the large sample sizes of many studies. However, when environmental heterogeneity is taken into account it becomes clear that infection specificity is not simply a binary trait but rather that it can depend on local resources [75], temperature [76], experimental approach [21], and dose [67]. For example, examination of *Cellulophaga baltica* strains and their associated phages isolated from coastal waters showed differences of up to 6 orders of magnitude in bacterial sensitivity to the same titer of phage [67]. This variation can be critical to predicting the impact of phages on bacterial communities, as fine-scale differences among bacterial genotypes or species in sensitivity to the same phage would mean a fitness advantage of one over the other. Therefore, studies that treat infection success as binary would fail to predict this apparent competition. Similarly, if broad host range phages are able to infect more hosts simply because they reach higher prevalence within the environment (given the greater number of available hosts), this specificity can be considered context-dependent. 

**Figure 3:**
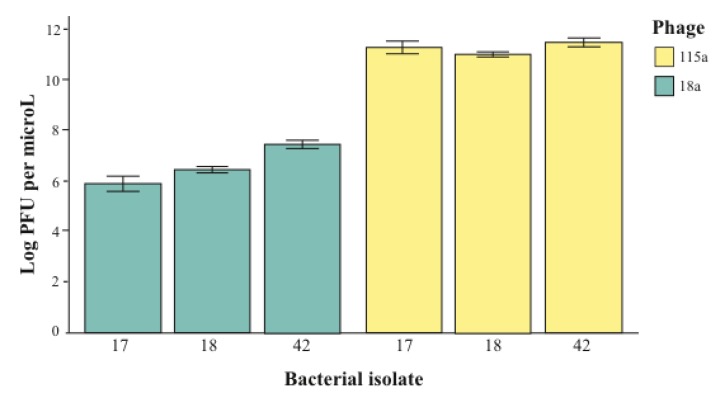
Results of the reciprocal cross-inoculation where the same phage inoculum was spotted in a dilution series onto lawns of each of three different *Erwinia sp.* bacterial isolates. The number of plaque forming units (PFUs) per microliter of inocula was measured for each cross, and the means across five replicates are shown on the Y-axis. Variation in PFU within a given inocula (*i.e.*, among the blue bars or among the yellow bars) represents variation in phage success across bacterial hosts. Note that the important comparison is within-phage variation, as between-phage variation reflects absolute differences in phage titer.

As an example, we measured whether the bacterial isolates used in our host range tests showed variation in sensitivity to the phages from the phyllosphere. To do this, we inoculated multiple hosts with a dilution series of the same phage inocula (and thus necessarily the same phage titer). In this way, we were able to compare the plaque forming units (PFUs) from each inoculum on the lawn of one bacterial host versus another. If the relationship between phage titer and phage infection success for a given inoculum were the same regardless of the host being tested, we would expect to find no significant difference in PFUs across hosts. On the other hand, if different measures of PFUs are found for the same phage inoculum across different hosts, this would suggest that resistance is a quantitative trait, and therefore that host range may be dose-dependent. Overall, we found a significant interaction between phage inoculum and bacterial strain on the PFU per μL observed across 5 replicate dilution series (general linear model with log PFU as a response variable and phage and bacteria as explanatory variables; F_5, 156 _= 3.25, p < 0.01). Furthermore, when we included only the bacteria-phage combinations for which the test was fully reciprocal (*i.e.*, each of the two phages were infective to the same three hosts), we again found this interaction effect (Figure 3; F_1, 29 _= 8.03, p < 0.01). We later tested whether the density of bacterial cells in the soft agar overlay could explain this result and were able to rule out this possibility, as PFU was not correlated with bacterial density within the ranges used. This suggests that direct competition between these three bacteria in the presence of either of these phages is likely to be biased towards the least susceptible, even though all three are within the host range of the phage, and reinforces the idea that infection is not a binary trait. However, given that all bacteria were susceptible overall, these results suggest that dose will only affect the binary outcome of host resistance when phage titer is very low. Moving forward, researchers should consider whether bacterial resistance to phage should be considered a quantitative or qualitative trait with regard to their specific question of interest. For example, if experimental evolution lineages are being compared to a control treatment of the same bacterial strain, resistance might be treated as a binary trait to aid in statistical comparison. In this case, the only caution would be in interpreting a negative result such as lack of a cost of resistance, as the approach may have lumped different degrees of resistance into a single phenotype. On the other hand, resistance should be considered as a quantitative trait, varying for example across multiple environmental conditions or dose, if the goal is to make predictions regarding how phages will alter the competitive hierarchy of bacterial species in a community.

## 3. Conclusions

We set out to highlight the importance of understanding phage host range as a key factor in predicting (i) how phages shape microbial communities, (ii) when genes encoding virulence and toxins might be transferred among bacterial species, and (iii) the potential success of phages as therapeutic agents against bacterial pathogens. Recent advances in experimental evolution techniques and comparative genomics have given us important new insight, including that phage host range can be altered by single mutations [44], but is often the result of complex epistatic interactions among mutations [45], and that selection can lead to increased host range [42], but that this increase often carries a significant fitness cost [36,38,77]. Studies of the host ranges of natural phages have demonstrated a high variability of specificity, ranging from phages with extremely narrow ranges of hosts within a single species to those that can infect bacteria across genera. A key new insight comes from comparison across studies and systems, and suggests that bacteria-phage networks tend to be statistically nested [18,70]. However, our understanding of these networks remains limited, as very few whole community analyses have been completed and, importantly, estimates of phage host range are only as good as the reference panel against which they’ve been tested. Furthermore, phage infection is rarely if ever a binary trait, and therefore studies that measure infectivity at a single dose, and/or under a single environmental treatment, are likely to miss key aspects of phage specificity. Moving forward, data from natural populations in which the community of both bacteria and phages can be properly represented and tested in a way that captures variation in infection success beyond a simple “yes/no”, will help elucidate the complex networks of interactions between phages and their bacterial host. 
